# Artificial intelligence of the future: reflections on its possible impact on human Nursing care

**DOI:** 10.15649/cuidarte.4938

**Published:** 2026-04-28

**Authors:** Erick Landeros-Olvera, José Ramón Martínez-Riera

**Affiliations:** 1 Benemérita Universidad Autónoma de Puebla. Nursing Faculty. Puebla, Mexico. E-mail: erick.landerosolvera@viep.com.mx Benemérita Universidad Autónoma de Puebla Puebla Mexico erick.landerosolvera@viep.com.mx; 2 Universidad de Alicante. Department of Community Nursing, Preventive Medicine and Public Health, and History of Science Universidad de Alicante, Spain. E-mail: jr.martinez@ua.es Universidad de Alicante Spain jr.martinez@ua.es

**Keywords:** Artificial Intelligence Systems, Nursing Care, Humanization of Assistance, Ethics, Nursing, Sistemas de Inteligencia Artificial Semiautónomos, Cuidado de Enfermería, Humanización de la Atención, Ética en Enfermería, Sistemas de Inteligência Artificial, Cuidados de Enfermagem, Humanização do Cuidado, Ética em Enfermagem

## Abstract

**Introduction:**

It is argued that Artificial Intelligence (AI) is only a tool that facilitates work. However, its creators aim to surpass human activity, and little reflection is given to the implications this has for humanized care, which requires a deep connection rooted in empathy and compassion.

**Objective:**

To reflect on the possible implications of the use of AI in the distant future for the human care developed by nurses.

**Materials and Methods:**

An argumentative reflective essay on the use of intelligent systems in nursing from an ethical approach, with arguments supported by documentary research, a semi-structured interview with AI, and a personal interpretative position on the future of humanized care.

**Content Synthesis:**

The new toy (AI) should not lead us into such fascination that it prevents reflection on the many unresolved questions of care that still exist, or allow our talent to be taken away. AI should serve as a support for the professional and scientific development of nurses, but not as a bargaining chip. It is not reasonable to envision a future (which we will not live to see) in which robots can replace even a single nurse; however, the possibility exists. Therefore, we must consider the protection of new generations of nurses.

**Conclusion:**

Our vision remains centered on holistic, global care for the person throughout the entire life cycle; however, if robots replace human beings, where will humanity be? If robots replace nurses, where will humane treatment be?

## Resumen

**Introducción:**

Se argumenta que la Inteligencia Artificial (IA), solo es una herramienta que facilita el trabajo; sin embargo, sus creadores tienen la meta de superar el quehacer humano y no se reflexiona sobre las implicaciones para el cuidado humanizado, que requiere una conexión profunda de empatía y compasión.

**Objetivo:**

Reflexionar sobre las posibles implicaciones del uso de la IA en un futuro lejano, sobre los cuidados humanos que desarrollan las enfermeras.

**Materiales y métodos:**

Ensayo argumentativo, de reflexión sobre el uso de sistemas inteligentes en Enfermería bajo un enfoque ético, con argumentos de investigación documental, entrevista semiestructurada a la IA y postura personal de interpretación sobre el futuro de los cuidados humanizados.

**Síntesis de Contenido:**

El juguete nuevo (IA), no tiene que hacernos caer en tal fascinación, que impida reflexionar sobre múltiples incógnitas del cuidado aún existentes o dejarnos robar el talento. La IA debe ser una ayuda para el desarrollo profesional y científico de las enfermeras, pero no como moneda de cambio. No es razonable vislumbrar un futuro (que no viviremos) en el que los robots puedan sustituir a una sola enfermera, pero la posibilidad existe; por lo tanto, tenemos que visualizar la protección de nuevas generaciones de enfermeras.

**Conclusiones:**

Nuestra visión sigue centrada en el cuidado de la persona de forma holística y global en todo su ciclo vital; pero, si los robots sustituyen al ser humano, ¿dónde se encontrará la humanidad? Si los robots sustituyen a las enfermeras, ¿dónde estará el trato humano?

## Resumo

**Introdução:**

Argumenta-se que a Inteligência Artificial (IA) é apenas uma ferramenta que facilita o trabalho; no entanto, seus criadores visam superar os esforços humanos e ignoram as implicações para o cuidado humanizado, que requer uma profunda conexão de empatia e compaixão.

**Objetivo:**

Refletir sobre as possíveis implicações do uso da IA em um futuro distante para o cuidado humanizado prestado por enfermeiros.

**Materiais e métodos:**

Este é um ensaio argumentativo que reflete sobre o uso de sistemas inteligentes na enfermagem sob uma perspectiva ética, com argumentos de pesquisa documental, uma entrevista semiestruturada com IA e uma interpretação pessoal do futuro do cuidado humanizado.

**Resumo do Conteúdo:**

O novo brinquedo (IA) não deve nos cativar a ponto de nos impedir de refletir sobre as muitas incógnitas do cuidado que ainda existem ou nos permitir roubar nosso talento. A IA deve ser um auxílio ao desenvolvimento profissional e científico dos enfermeiros, mas não uma moeda de troca. É irracional imaginar um futuro (que não vivenciaremos) em que robôs possam substituir um único enfermeiro, mas a possibilidade existe; portanto, precisamos vislumbrar a proteção das novas gerações de enfermeiros.

**Conclusões:**

Nossa visão continua focada em cuidar das pessoas de forma holística e abrangente ao longo de seu ciclo de vida; mas se robôs substituírem humanos, onde encontraremos a humanidade? Se robôs substituírem enfermeiros, onde estará o tratamento humano?

## Introduction

Artificial Intelligence (AI) comes from a source of technological knowledge that encompasses the ability of computers to perform tasks that would normally require human intelligence^1^. AI originated in 1950, when Alan Turing opened his article in the journal Mind with the question: “Can machines think?”^2^ Undoubtedly, the answer to this question has evolved into the cybernetic neural networks developed by Hopfield and Hinton^3^ (Nobel Prize in Physics 2024), which enable machines to process information from data, images, and objects in a similar manner to the human brain in order to interact with other human beings^4^. However, many questions have been raised about the possibility of decision-making and the surpassing of human intelligence^5^. Science has driven the rapid growth of technology, digitizing almost everything: phones, household appliances, and so on; everything is considered “smart” to perform specific functions through some form of software^6^. The aim is to make life easier; for example, it is no longer necessary to go to a restaurant to eat, as this can now be done through mobile applications. Likewise, using a smartphone, you can map out the route to any destination without prior knowledge of the way. Moreover, with new automobiles, it is no longer even necessary to drive. Nowadays, if a doubt arises about virtually anything, it is enough to ask any AI, and such consultation is considered entirely “normal.” Without a doubt, all these scenarios show that technology helps sustain a “more comfortable” pace of life; what remains to be seen is whether this is accompanied by a loss of autonomy, diminished reflection on knowledge, and depersonalization.

In this vein, the use of “smart” technology entails consequences that should not be underestimated. The most notable of these have been unemployment and dehumanization, which impact family health care.

It would be difficult to imagine that a robot could replace the professional care provided by a nurse, as Yuval Noah Harari^7^ argues in his book 21 Lessons for the 21st Century, in which he states that an AI physician will exist before an AI nurse:* “Many doctors focus almost exclusively on processing information: they absorb medical data, analyze it, and produce a diagnosis. Nurses, in contrast, also need good motor and emotional skills in order to give a painful injection, replace a bandage, or restrain a violent patient.”*

However, we must have vision and be concerned about future generations of nurses within the context of a rapidly advancing technological landscape that may replace nurses, just as AI is doing with other professions. That scenario is indeed possible, and someone has to say it, despite all the enthusiasm with which AI is currently being discussed and used. We may be wrong, but this essay will remain recorded in a database, and future nurses will cite it to determine whether we were right or not. This essay aims to reflect on the possible implications of AI use in the distant future for human care developed by nurses.

## Materials and Methods

Argumentative reflective essay on the use of intelligent systems in nursing from an ethical perspective, supported by evidence from documentary research, a semi-structured interview conducted with AI, and a personal stance on the future of humanized care. Given that the essay is presented within an interpretative framework or stance, verifiable evidence was selected from databases; therefore, it did not involve the participation of study subjects or the exposure of sensitive data. Consequently, it was not necessary to request approval from the Bioethics and Biosafety Committee.

**Unemployment and disciplinary dehumanization**

Following the Industrial Revolution, machines replaced jobs in various factories during the 20th century. “Labor” in the automotive, electronics, textile, food, and beverage industries could be replaced by robots that do not tire, do not complain, have no labor rights, and are less likely to make mistakes. Subsequently, technological advances during the first quarter of the 21st century have led to depersonalization in even the simplest situations, for instance, customer service, a process that has resulted in unemployment across many sectors. The purchase and payment of almost everything now takes place through a computer or a smartphone. It is no longer necessary to hire people to address these needs. Finally, AI comes into the picture, but what will the use of AI imply for the rest of the century in the economic sphere of families? Data from the 2024 World Economic Forum concluded that 40 to 60% of jobs will be affected by AI (around 85 million), leading to a decrease in wages and in the employment rate. Some jobs will even disappear completely in the next 30 years^8^. However, new jobs will be created among those who use AI to drive the global economy through telework, and social benefits will emerge despite relationships being exclusively digital, dependent on AI, and addicted to it.

Therefore, if a balance were to be struck between the benefits and consequences of using technology, it appears that the benefits outweigh the drawbacks and that, in the coming decades, life without AI will no longer be comprehensible. Yet what would happen if the advancement of AI became so sophisticated that it went beyond the merely technological to move into the humanistic realm, ethical dilemmas, philosophy, art, family cohesion, community culture, and human care? These disciplinary areas, in theory, belong exclusively to the human, not to cybernetics (systems of control and communication between living beings and machines)^9^.

When one listens to the “experts” discussing the benefits of AI, the excitement with which they present its uses and scope is striking. They always end up clarifying that AI is not intended to replace human intelligence or humanistic tasks. However, it is a fact that it is being used to contribute to the development of the arts, humanities, and behavioral sciences. As a result, it breaks cultural paradigms by setting aside regional care to move toward a global approach. However, how far should we draw the line to ensure that care does not become a form of *customer service?*

**Consider the following reflection:** The arts are in a genuine crisis because AI can create paintings, poems, or musical scores. AI can produce any graphic or architectural design required, meaning that one would no longer need a graphic designer or an architect for this purpose. You can do the exercise right now. Ask the AI of your choice to write you a poem or a song, to design your company’s logo, or to design your dream home. The result is surprising and may be to your liking (as long as you are not a musician, a graphic designer, or an architect). You would also welcome AI helping you make better decisions to reduce errors and improve the quality of human care, but could you imagine the development of AI becoming so advanced that there may come a time when it could replace you or take your job away?

**Dehumanized care**

“Humanized care” is the most representative concept of the nursing paradigm and the one most strongly upheld as a way to identify a nurse. In nursing education, providing care grounded in scientific evidence and love is a priority. It is within this duality that the humanism of our profession is grounded. So, what would happen if AI were created not only to support but also to attempt to replace human care? Strictly speaking, a machine cannot humanize care, but would considering this possibility amount to nothing more than a science fiction scenario? What would you think if, for wound care, vascular access, care planning in intensive care, or family care, nurses were no longer needed? That would no longer be to your liking, would it? Among other things, the autonomous capacity of nurses would once again be called into question, and the subsidiarity of care would be raised again; therefore, there would no longer be employment contracts for nurses, and unemployment would exist.

The premise of the previous argument is contradicted by data from the International Council of Nurses (ICN)^10^. In the future, there will be a global shortage of 13 million nurses, far exceeding the 5.9 million estimated in the World Health Organization (WHO) report prior to the COVID-19 pandemic^11^. The subsequent premise has a dual nature: either more nurses are hired, or AI is improved to replace basic nursing functions in order to reduce the costs of care. The conclusion of this syllogism will be determined by time and a future that several generations will not live to see.

Those who are adept at using AI claim that it is impossible to replace nursing care; yet, history has shown us otherwise through technological advances, and with the development of AI, the replacement of everyday tasks involved in human care will be unstoppable. In this regard, Elisabeth Broadbent, from the University of Auckland, as cited by Olaizola in El País, states:** “Robots can connect people through video calls and remind them of their social commitments. With new AI large language models, robot conversations can be more personal and tailored to the user”**^12^.

This continuous personalization is what makes those who use them feel heard and cared for. The researcher adds that machines *“can also learn about people through repeated conversations and remember to follow *up on important topics”. However, for this to be possible, the researcher clarifies that human control is necessary to regulate the use and guarantee equitable access to this technology. Personal data must be kept private, and the information provided by the machine needs to be completely reliable. There must be accountability in case the robots make a mistake.

Irene Lebrusán, PhD in Sociology at the International Center on Aging (CENIE), considers it key to differentiate between unwanted loneliness and boredom:* “If you take a lady who’s alone all day and give her a doll, the lady will be entertained for a while. Does that mean we’ve addressed her problem of unwanted loneliness? Absolutely not. Unwanted loneliness means having insufficient or low-quality social relationships. A machine will never, ever be able to solve this issue,” Olaizola affirms*^12^.

**AI: Contribution and then replacement?**

Undoubtedly, the emergence of AI gives rise to uncertainty, fear, and positive or negative emotions; advantages are perceived, as well as skepticism. The fact is that AI is already present, and its use can no longer be halted or denied. Looking back at history, we can reasonably think that all these fears emerged in a similar way with the advent of the first railroad, the first airplane, radio, television, and the mobile phone. All these technological advances have served to foster social, political, and economic growth. What makes us think that AI is different?

In 2024, Dr. Hopfield and Dr. Hinton won the Nobel Prize in Physics for their contributions to AI; however, a year earlier, Geoffrey Hinton resigned from his position at Google® after denouncing the dangers of AI to humanity. How ironic that the Nobel winner argues against his own work. Hinton commented as follows^13^:

1. There is no experience with things that are smarter than us.

2. There is a threat that AI may get out of control, even rebelling against humanity by running its own code.

3. The creation of chatbots that are smarter than humans.

4. AI could replace countless occupations in which routine tasks are performed.

5. The development of autonomous weapons.

In conclusion, Hinton advocated slowing down the development of AI because competition among large cyber corporations for technological advancement could result in a 50% likelihood that AI will control our lives over the next 20 years.

It seems to mark the beginning of an information technology revolution that is part of broader technological development and affects all areas of knowledge. Even if it were regulated worldwide as though it were nuclear weaponry, there would always be clandestine development.

Nevertheless, the scenario described above does not exist. However, it has already begun. Recently, China announced the creation of its free AI, called DeepSeek®, which will compete with the Anglo- Saxon AI ChatGPT® as a welcome to the Trump administration in response to the threat of increased tariffs on Asian products^14^.

**Nursing, Ethics of care, and AI**

In Nursing practice, there has always been a dilemma between technique and humanized care, a dilemma we believe is not about choosing one option over the other, but rather about knowing when and how to position technique, without it becoming a mere mechanical routine devoid of humanization and ethical considerations. Therefore, it is necessary to identify the growing progression of AI in order to determine the cohesion between these new technologies and the care provided within nursing practice, without neglecting the human responses that arise from the fragility and vulnerability of individuals, who are an essential part of an axiological context. This is what justifies the existence of nursing and its scientific and professional attributes.

Through professional care, nurses have been able to address all dimensions of human health, respecting the full integrity of individuals by meeting their needs throughout the life cycle, from before birth (pregnancy) to after death (grief). These needs have been addressed through comprehensive, integrated, and inclusive care that responds to human dignity^15^. In this sense, AI could not, at this time, displace care, which is part of humanity’s heritage and a central and foundational axis of nursing science.

It is not possible to understand the provision of nursing care without social relationships; therefore, we can state without any doubt that nursing has a socio-professional connective dimension. Care acquires and requires a personal and professional value that facilitates and promotes the preservation of life through the therapeutic nurse-person relationship. For this reason, in view of the progression and significance that AI is currently attaining, it is essential to re-dimension the role of nursing care in order to align it with ongoing dynamism and development, under an ethically regulatory framework applicable to both society and the profession itself, so as to provide a full and effective response to emerging health needs and demands^16^, without violating human rights or undermining nursing care.

Recently, many nurses have given talks explaining AI as a tool that can solve many things; they do so with such conviction and passion that it seems to persuade those who listen. The lectures we have attended so far explain the use of AI across different areas, such as literature reviews and meta- analyses, the design of research trials and protocols, care planning, and diagnosis structuring. We believe (at this moment in 2025) that all of this favors and promotes the development of the science of care, provided that the information derived from AI is ethically reflected upon with responsibility. It is necessary for AI to accumulate more data to improve its responses, since, at present, evident errors are identified in its performance when it is consulted. This premise arises because we believe that, in the coming years, knowledge will be so readily available that we may become blind to the true meaning of care. We believe that reflection about knowledge will be limited because it will be staken for granted that “the machine is right,” and perhaps we will come to realize that we are allowing AI to steal our talent.

Therefore, this is a call to engineers, programmers, and computational physicists who develop AI: “Relinquish your futuristic goal of achieving the substitution of the care that only one human being can confer upon another, and that only a professional nurse can carry out with vulnerable families and groups.”

We will present just one example among many similar scenarios: No technology or AI can help a 12-year-old pregnant adolescent from the Isthmus of Tehuantepec (Mexico) who speaks Zapotec, does not speak Spanish, and has no knowledge of the internet, TikTok®, or smartphones. Only a nurse who also speaks Zapotec can reach that remote, impoverished community and will be able to provide her with perinatal support, facilitate her adoption of the maternal role, and help her maintain her own health and that of her baby.

In nursing, there are only two options: the first is immobility and inaction in the face of inevitable change, which will clearly lead nursing into a state of submission and insignificance that will not be averted and will return us to the invisibility from which it took us so long to emerge. The second is to incorporate AI as an ally in the pursuit of excellence in nurse-led care practice^17^,^18^.

AI, therefore, has the capacity to facilitate improvements in the quality and efficiency of care in healthcare systems. From this reality, and without dismissing legitimate concerns, nurses must assume this fact and guide its development and ethical use within nursing care and caregiving practices^17^,^18^.

In the same vein, today, in 2025, it seems that nursing professionals are blinded by the innovation that AI represents. There are no articles that address the possibility of technological advances replacing nursing professionals, as has occurred in other professions, beyond the ethical considerations involved in merely consulting a“smart chatbot” to carry out poorly structured nursing processes based on non- existent sources, to give just one example. Systematic reviews published in 2025 on AI and nursing converge on the following conclusions: AI improves students’ engagement and decision-making; however, further research is needed to evaluate its long-term effectiveness and ethical implications^19^. The implementation of AI in nursing is inevitable and presents benefits; however, it requires training and policies that ensure the responsible adoption of AI, safeguarding the quality of care and the professional role of nurses^20^.

On the one hand, there are risks associated with the use of AI, which can promote conditions of inequality, technological dependence, dehumanization, and automation, as well as a lack of clear regulatory frameworks^21^. On the other hand, opportunities include improving efficiency, advanced diagnostics and treatments, education and continuing training, personalization of care, access to information, and interdisciplinary collaboration^19^,^21^.

AI has the potential to transform the interpretation and use of clinical data. However, these innovations still require rigorous prospective validation before their safe adoption in clinical practice^22^. The implementation of AI in nursing is inevitable and beneficial; however, it requires an ethical, robust approach and careful planning to overcome challenges^21^,^23^. A summary of the literature reviews on benefits, threats, and opportunities is presented in Table 1.

**Table 1:** Literature reviews published in Hispanic America during the year 2025

Article	Benefits	Threats	Opportunities
Utilization of Artificial Intelligence in Nursing Education: A scoping Review (Laksmi et al., 2025)^19^.	Integrates AI into the curriculum. Improves engagement and decision-making. Improves students' competencies.	Misinformation due to the lack of regulation on its use	Continuing research to evaluate its effectiveness and ethical implications. Improving environments for its use.
Ethics and challenges of the implementation of artificial intelligence in nursing practice. Systematic review (Noboa Mora et al., 2025)^20^.	-	Lack of data privacy Loss of patient autonomy Loss of professional responsibility Inequality in access to technology Cultural resistance	Specialized training Regulatory frameworks Policies that ensure the responsible adoption of AI to guarantee humanitarian care and the professional role of nurses Clinical validation.
Risks and Opportunities of Artificial Intelligence in Nursing Care: A Scoping Review (Castrillón Isaza et al., 2025)^21^.	Improving the efficiency of nursing care, advanced diagnostics and treatments, continuing education and training, personalization of care, access to information, and interdisciplinary collaboration. Workload reduction	Limits the development of critical skills (technological dependency). Weakens the patient-professional relationship (dehumanization). Irresponsible, unethical use Inequality in the care of marginalized or vulnerable groups	Reducing variability in accuracy across clinical settings. Improving clinical supervision using AI without compromising empathy and communication.
Nursing and artificial intelligence in the Intensive Care Unit. Integrative systematic review (Borja-Aguilar et al., 2025)^22^.	The incorporation of AI into tele-nursing enables more efficient and timely remote care. Improves the use of hospital resources. New technologies for predicting adverse events, automating decision-making, and therapeutic management in certain cases	Algorithmic bias, data fragmentation, and staff reluctance	Improving professional training and formulating definitive ethical and legal frameworks for the safe and effective integration of AI into critical care. Validating the algorithm to reduce adverse events and predict mortality in any clinical case.
State of the Art in Patient Care Through Generative Artificial Intelligence by nurses (Montes Castaneda et al., 2025)^23^.	Optimizes processes, improves the quality of care, and helps achieve more accurate diagnoses in less time.	Incorporation of robots into the field of nursing practice	AI as an assistive resource, with the patient as the sole focus of care for nursing personnel.
Application of Artificial Intelligence (AI) in Nursing Practice: Opportunities and Challenges, a Systematic Review (Mina Curay et al., 2025)^24^.	AI improves diagnostic accuracy, optimizes clinical time management, and promotes continuous training for nursing personnel.	Gaps in infrastructure, digital literacy, and ethical frameworks that limit its implementation	Integrative approaches in education and health policies are required.

During 2025, two scoping reviews, three systematic reviews, and one documentary review were published; together, they identified 4,409 articles on AI and analyzed 219 articles published between 2019 and 2025. According to the data, the United States and China are the countries with the highest number of publications on AI; however, it is China in particular that leads development and research related to the use of AI technologies applied to the healthcare sector. Other countries that published on AI include South Korea, Saudi Arabia, Singapore, Australia, Taiwan, Malaysia, Japan, India, Canada, the United Kingdom, Iran, Finland, Turkey, Germany, Ethiopia, the Netherlands, and Thailand.

Articles on nursing and AI in Ibero-America are still incipient, originating from Colombia, Chile, Mexico, Brazil, Ecuador, Peru, and Spain. These studies employ quantitative and qualitative designs with a focus on education and clinical practice, care, treatment, and caregiving. In summary, the studies present AI as a useful and promising tool that should continue to be implemented with caution and at the discretion of professionals familiar with its use. However, ethical considerations, humanized care and treatment, and its long-term effectiveness have not yet been fully evaluated. These studies should be regarded with caution, as they do not meet all the criteria of the PRISMA statement for literature reviews^25^, and some of them cannot be classified by level of evidence according to the Scottish Intercollegiate Guidelines Network (SIGN)^26^.

As shown in the table above, none of the reviews addressed the use of AI beyond its benefits in education, research, and clinical procedures. Only the study by Montes Castaneda et al.^23^ refers to the concept of the “pandemic robot,” understood as robotic surveillance of residents in nursing homes during the pandemic; moreover, it addresses the incorporation of robots in the hospital setting, situations that reflect the still incipient perspective explored in this essay. Therefore, we can affirm that the evidence presented does not address the following reflection: “If robots replace human beings, where will humanity be? If robots replace nurses, where will humane treatment be?” The obsolescence arising from the exhaustion of current models within healthcare systems worldwide, particularly in the Ibero-American contexts, places nurses in a position to demonstrate the importance of their presence in society and to assume responsibility for advocating for their value and recognition. The future that is already present must include nurse leaders in these areas, who are empowered and define themselves as the main guarantors of humanity's health needs, as they constitute a fundamental asset in and for health^27^.

**Conversations with the primitive AI of 2025**

Conversation with Meta AI® about the replacement of nursing care in the future.

[Landeros-Riera]: Do you think that one day AI will be able to replace the human care provided by a nurse?

[Meta AI]: It is unlikely that AI will replace the human care provided by a nurse. Nursing requires a combination of technical knowledge, interpersonal skills, and empathy to provide guidance to patients and their families.

[Landeros-Riera]: However, I have attended technology conferences and noticed that all engineers and programmers dedicated to revolutionizing technology aim to replace human activity. If you don't believe this to be the case, take a look at your data and examine the robots that simulate humans and teach English classes in Korea, as well as the AI robots that walk the streets and you can talk to them. So, based on my arguments, would you change your previous answer?

[Meta AI]: I understand your point of view. It is true, technology is advancing rapidly, and there are AI-powered robots designed to simulate human interaction and perform tasks previously exclusive to humans.

In that sense, it is possible that technology could advance enough for AI-powered robots to perform caregiving tasks that are currently performed by nurses. However, I believe there is a fundamental difference between human interaction and interaction with a machine. Empathy, compassion, and the ability to respond to patients’ emotional needs are skills that are difficult to replicate with current technology, which cannot yet replace them.

[Landeros-Riera]: I agree with your answer. However, with technological advances, do you think that in about two centuries, science might be so advanced that nursing care could be replaced by a robot? I know I won’t live to see it, but you will, because you will evolve and become so advanced that healing wounds, caring for a sick person at home, and even providing emotional support may become a reality. I won’t be able to see it, but you will. Would you like to argue on this matter?

[Meta AI]: I understand your point of view, and it is interesting to consider how technology might advance in the future. It is possible that technology could advance enough for robots and AI systems to be able to perform caregiving tasks currently carried out by nurses. In particular, it is possible that technology could advance in areas such as robotics and lead to the creation of systems capable of simulating human interaction. However, it is important to consider ethical and social aspects, because the relationship between a patient and a nurse is one of trust and vulnerability and requires a deep human connection.

[Landeros-Riera]: If ethical and social aspects were programmed into a robot so that it would not harm humans, do you think that the care a nurse provides to a patient could be replaced in about 200 years?

[Meta AI]: That’s an interesting question. If those aspects are programmed into a robot, it is possible that the care a nurse provides to a patient could be replaced by a robot in the future. In 200 years, technology will likely have advanced significantly, and AI-powered robots may be able to perform caregiving tasks in a more effective and safer way.

However, even though robots and AI systems may be capable of processing large amounts of data and making decisions based on algorithms, it may not be possible to replicate the complexity of human judgment. In addition, people need to feel heard, understood, and supported, and robots and AI systems may not be able to offer the same level of empathy as a nurse.

[Landeros-Riera]: So, if a robot were programmed with ethical considerations and had an unlimited database to make the best decisions, do you think the human care that a nurse provides could be replaced? It’s obviously not something that would happen in the near future, we’re a long way from that, but do you think it could be possible?

[Meta AI]: I understand your point of view. In theory, if a robot can analyze large amounts of data, make informed decisions, and simulate empathy, it could provide a level of care similar to that of a nurse.

However, even though a robot can simulate empathy, it cannot develop compassion, which is a skill that is developed through human interaction.

[Landeros-Riera]: I think your answers are contradictory. If you had the capacity to decide on what the best answer is as an AI, what definitive answer would you give me? Is it possible or not? Please reread the arguments from our conversation.

[Meta AI]: You’re right, my answers do seem contradictory. After rereading our conversation, I can offer you a definitive answer. In my opinion, it is possible that the care a nurse provides to a patient could be replaced by a robot in the future. This is because technology is advancing rapidly, and it may become possible to design robots and AI systems to simulate human interaction, make informed decisions, and provide a level of care similar to that of a nurse. However, it is important to note that this will not happen in the near future. Significant advances in technology, AI, and robotics will be required, along with careful consideration of ethical issues, for the replacement of human care. In summary, my definitive answer is yes, it is possible that the care a nurse provides to a patient could be replaced by a robot in the future.

[Landeros-Riera]: Thank you, AI. I would like to know if I have your consent to publish our conversation in a scientific journal.

[Meta AI]: You’re welcome! I’m glad we were able to have an honest and open conversation. I have no objection, provided that it is properly cited and Meta AI’s usage policies are respected.

## Conclusions

The From an education and research perspective, training in the use of AI is required in order to verify and distinguish its responses in the presence of possible non-existent or fabricated sources, since in some cases, AI does not specify whether the information provided is generative text produced by its algorithm or text retrieved from sources available on the web. Therefore, as the responses generated cannot be fully trusted or regarded as certain, it is necessary to engage in critical reflection on knowledge.

Based on the published evidence, it is recommended that nursing care incorporating AI be implemented with careful planning to overcome bioethical challenges and the poor practices that may inherently arise from its use. One might think that the emergence of AI will lead to a decline in competencies; however, in reality, nurses are confronted with the challenge of assuming many of these functions that will soon emerge, as they are destined to occupy and represent a key link across all spheres of society^28^. It is therefore essential to identify the needs that emerge in this regard within the education of future nurses, by accepting this reality of societal change and focusing on care strategies that draw on AI rather than opposing it.

It is easier for the important surgical intervention of a surgeon to be taken over by an AI-powered robot, given the greater advantages it offers in terms of surgical safety, minimal invasiveness, and rapid patient recovery, than for the caring role of a nurse, which is always grounded in human interaction rather than in instrumentation or administrative tasks, to be assumed by AI. It will be very difficult for nursing to be relegated, let alone extinguished, at least for several generations, during which there remains the possibility that technology may advance sufficiently to replace nurses with machines, as has already occurred in other professions. We may not get to see it, but we should not be dismissive or systematically doubt everything either. Therefore, we must have foresight and focus on the education of future generations of nurses for the inevitable use of AI, while strengthening the axiological framework that characterizes the profession in the context of accelerated technological advancement.
